# Does lower dose pilocarpine have a role in radiation-induced xerostomia in the modern radiotherapy era? A single-center experience based on patient-reported outcome measures

**DOI:** 10.1007/s00405-024-08616-x

**Published:** 2024-04-04

**Authors:** Dilek Gül, Beste M. Atasoy, Ece Ercan, Zilan Başkan, Kıvanç Bektaş Kayhan

**Affiliations:** 1 S.B.-Marmara University Pendik Education and Research Hospital Radiation Oncology Clinic, Istanbul, Turkey; 2 Department of Radiation Oncology, School of Medicine, Marmara University, Istanbul, Turkey; 3 Department of Oral and Maxillofacial Surgery, İstanbul University Faculty of Dentistry, Istanbul, Turkey; 4 Marmara Üniversitesi Pendik EAH Radyasyon Onkolojisi Kliniği, Fevzi Çakmak Mah. Muhsin Yazıcıoğlu Cad. No: 8, 34899 Istanbul, Turkey

**Keywords:** Lower dose, Patient-reported outcome measurement, Pilocarpine, Radiotherapy, Xerostomia

## Abstract

**Purpose:**

This study aims to investigate the efficacy of lower dose pilocarpine in alleviating late dry mouth symptoms in head and neck cancer patients received radiotherapy.

**Methods:**

Eighteen head and neck cancer patients experiencing persistent dry mouth were enrolled in this study. All participants started pilocarpine treatment a median of 6 months post-radiotherapy. Initially, patients received pilocarpine at 5 mg/day, with a gradual increase to the recommended dose of 15 mg/day. A Patient-Reported Outcome Measurement (PROMs) questionnaire assessed symptoms’ severity related to hyposalivation.

**Results:**

All patients reported symptomatic dry mouth above grade 2 before starting the medication. Pilocarpine treatment continued based on patients’ self-assessment, with a median duration of 12 months (range, 3–36 months). The median daily maintenance dose was 10 mg (range, 5 to 20 mg). Total PROMs scores significantly decreased following medication, from 13 points (range 7–18 points) to 7 points (range 4–13 points) (*p* = 0.001). Significant improvements were observed in questions related to dry mouth (*p* < 0.001), water intake during eating (*p* = 0.01), carrying water (*p* = 0.01), taste (*p* < 0.001), and water intake during speech (*p* < 0.001). Initial and maintenance doses of pilocarpine were lower, and the duration of pilocarpine usage was shorter in patients treated with intensity-modulated radiation therapy compared to conformal radiotherapy (12 months vs. 25 months, *p* = 0.04).

**Conclusion:**

Pilocarpine may be considered at doses lower for late-term dry mouth. With modern radiotherapy techniques effectively preserving the parotid gland, short-term use may be recommended in these patients. Future studies may enhance the development of a more robust patient selection criteria model.

## Introduction

Head and neck cancer is the sixth most common cancer worldwide and radiotherapy (RT) plays an important role in treatment as a part of multidisciplinary modalities [1]. The main aim of RT is to deliver the maximum dose to the tumor with minimal toxicity on neighboring healthy tissues [2]. Despite that, radiation-induced dry mouth is one of the common side effects in head and neck cancer patients both for early and late periods [3–5]. Salivation quantity and quality frequently get worse, and this effect can lead to progressed dental problems, recurrent oral infectious, compromised mucosal integrity, impaired speech, chewing, swallowing, sense of taste and difficulty in maintenance of nutrition. Patients often complain of chronic oral pain and mouth burning [5, 6]. All these symptoms and complaints decrease the quality of life in head and neck cancer patients [6].

Modern radiotherapy as intensity-modulated radiotherapy (IMRT) tries to reduce the side effects [7, 8]. However, radiation-induced toxicity is still challenging to patients’ quality of life [9]. Comparing to conventional radiotherapy, IMRT technique helps to decrease early and late side effects [10]. Especially, dry mouth incidence and patients’ compliance significantly decrease with IMRT technique by parotid gland sparing irradiation [11].

Meanwhile, radioprotectants are also used for xerostomia both for prevention and treatment [12–17]. Phase III trials showed that amifostine showed a significant radioprotection effect for salivary gland in head and cancer patients [14, 15]. However, there are some critics for this protectant in clinical usage in terms of toxicity and economic analysis [13, 15, 17, 18]. There are different options to reduce radiotherapy-induced dry mouth and symptomatic relief in post-radiotherapy patients such as sialagogue medications, saliva substitutes, acupuncture, hyperbaric oxygen, and salivary gland transfer [3, 4, 19, 20]. Pilocarpine is a cholinergic parasympathomimetic agent known before three-dimensional radiotherapy. The drug increases salivary secretion by stimulating cholinergic muscarinic receptors on the surfaces of exocrine glands. Oral pilocarpine therapy during and after radiotherapy is approved in many countries for the treatment of radiotherapy-induced xerostomia in the head and neck region [21–24]. A multicenter, randomized, double-blind, placebo-controlled study showed that pilocarpine was used for the systematic treatment of xerostomia after radiotherapy with best results lasting 8–12 weeks [23]. However, pilocarpine has serious side effects such as nausea, headache, frequency, vasodilatation, dizziness, dyspepsia, and sweating. The severity and incidence of these side effects is dependent on the recommended dose of pilocarpine medication (15 mg/day) [20, 21]. In some studies, successful results have been obtained in reducing dry mouth with cevimeline like pilocarpine [25].

This study aimed to investigate the symptomatic relief of dry mouth treated by lower dose pilocarpine in long-term usage after conformal or intensity-modulated radiotherapy in head and neck cancer patients.

## Materials and methods

This study was done with ethical committee permission of our faculty (Number: 09.2011.0147). There were 18 head and neck cancer patients (nasopharynx, *n* = 8; oral cavity, *n* = 7; tonsil, *n* = 3) agreed to participate in the study voluntarily. Patients received three-dimensional conformal (3DCRT) (*n* = 11) or intensity-modulated (*n* = 7) radiotherapy (IMRT). The mean planning target volume dose of primary tumor were 60 Gy (range 36 to 68 Gy) for 3DCRT and 62 Gy (range, 36 to 70 Gy) (*p* > 0.05). Both side of submandibular and other minor salivary glands were involved in the radiotherapy planning target volumes due to lymphatic irradiation. Meanwhile, the salivary glands (parotid and submandibular) were countered to calculate the radiotherapy doses in treatment planning system. The first dose of pilocarpine was started as single dose of 5 mg/day and was increased to recommended dose (15 mg/day) within couple of days. First dose of pilocarpine was especially recommended to take before sleeping. Depending on the patients’ symptomatic relief and requirement, the maximum pilocarpine dose was increased to 20 mg/day. If any toxicity more than grade 2 occurred, the pilocarpine dose was decreased or quitted by physician. Otherwise, dose increments or discontinuation of the treatment were carried out based on the patients’ decisions. Toxicity assessment was done according to CTC v3.0 [26]. Table 1 shows the semi-quantitative patient-reported outcome measurement (PROMs) questionnaire for dry mouth evaluation that was also used as in our previous study [27]. The minimum score was 0 and the maximum score was 18. Higher scores revealed the severity of dry mouth. Patients completed this form at the beginning and during maintenance therapy or at the end of pilocarpine usage. At the time of starting the medication, quality of life (QoL) questionnaires of European Organisation for Research and Treatment of Cancer (EORTC)-C30 and EORTC-HN35 Turkish were performed for each volunteer [28–30]. Data of before and after medications were compared based on the individual volunteers. Group means were used for statistical analysis and *t*-test was used for the difference between the beginning and the end of therapy. Differences according to treatment planning techniques was also evaluated with *t*-test. Significance level was set to *p* < 0.05.

**Table 1:** Patient-reported outcome questionnaire for dry mouth

Questions	A	B	C	D
Have you had dry mouth during the day?	I have not any dryness	Mild	Moderate	Severe
Do you feel the need to drink water while eating?	None	A small amount of	In every bite	I cannot take any solid food without drinking water
Do you carry water with you to wet your mouth?	None	Occasionally	Frequently	Always
Do you taste the food you eat?	I can taste all foods	I cannot taste some of the foods	I cannot taste most of the foods	I can taste no food
Do you need to wet your mouth during talking?	None	Yes, I need to wet my mouth if I talk too long	Yes, I need to wet my mouth in every talk	I need to wet my mouth even I say a few words
Do you have to examine by your dentist due to oral/dental problems following radiotherapy?	None	One or two times	Regularly	I had a dental problem required intervention
Score	0	1	2	3

## Results

The median time between radiotherapy completion and initiation of pilocarpine was 6 months (range 3 to 24 months). All patients had symptomatic dry mouth more than grade 2 at the beginning of pilocarpine medication. The treatment continued due to patients’ self-requirement with the median duration of 12 months (range, 3 to 36 months). Pilocarpine-related grade 2 and more toxicities were observed in 12 (66%) patients. Three (16.6%) patients discontinued the drug due to side effects of asthenia, tremor, relaxation, polyuria, headache, and flushing in 3 to 6 months. Dose reduction was made in eight (44.4%) patients, whereas three patients requested to receive pilocarpine to increase the dose 20 mg/day. The median maintenance daily dose was 10 mg (range, 5 to 20 mg). There were only 22.2% patients who received the recommended dose of 15 mg/day in maintenance.

In the QoL questionnaire, at the beginning, the results showed that mean dry mouth score was 49.9 points, sticky saliva was 51.8 points, difficulty in swallowing was 23.5 points, and lack of taste was 18.5 points. The median total PROMs scores were declined significantly following the medication from 13 points (range 7–18 points) to 7 points (range 4–13 points) (*p* = 0.001). For questions of having dry mouth (*p* < 0.001), requirement of drinking water during eating (*p* = 0.01), carrying water (*p* = 0.01), taste (*p* < 0.001) and drinking water during speech (*p* < 0.001) were significantly better following medication. Xerostomia intensity was also significantly declined after medication (grade 2, range grade 2 to 3 vs. grade, range 1 to 2) (*p* < 0.001). Individualized details and results of 18 patients are summarized in Table 2.

**Table 2:** All results according to the patients and treatments characteristics

Patient no.	Radiotherapy technique	Duration of pilocarpine (month)	Initial dose (mg)	Maintenance dose (mg)	Initial PROMs questionnaire scores	Maintenance PROMs questionnaire scores
1	Conformal	30	15	10	14	6
2	Conformal	36	20	10	12	4
3	IMRT	3	5	1.25	8	5
4	Conformal	10	10	20	13	4
5	Conformal	36	20	10	14	5
6	IMRT	16	10	10	9	6
7	Conformal	24	15	10	18	11
8	Conformal	6	10	5	9	4
9	Conformal	6	10	10	9	7
10	Conformal	6	10	15	12	8
11	IMRT	4	10	15	10	5
12	IMRT	6	10	10	7	3
13	IMRT	12	15	15	16	11
14	IMRT	12	15	15	17	10
15	Conformal	34	15	10	15	5
16	Conformal	36	15	10	17	11
17	Conformal	25	15	15	18	13
18	IMRT	12	15	15	17	10

*PROMs* patient-reported outcome measures, *3DCRT* 3D conformal radiotherapy, *IMRT* intensity-modulated radiotherapy

Analysis due to radiotherapy techniques are summarized in Table 3. Radiotherapy doses for target volumes (primary tumor and lymphatics) were not different in 3DCRT and IMRT groups. Both parotid glands received significantly lower doses (median 28.5 Gy, *p* = 0.023 and 28.3 Gy, *p* = 0.023). However, submandibular glands received almost the same doses of radiotherapy targets (median 52.3 Gy and 50.2 Gy) regardless of treatment techniques. Initial and maintenance doses of pilocarpine medication were lower in IMRT patients. Moreover, the duration of pilocarpine usage was less in IMRT group (12 months vs. 25 months, *p* = 0.04).

**Table 3:** Comparing the results according to radiotherapy techniques

	3DCRT (*n* = 11)	IMRT (*n* = 7)	*p*
	Median (range)		
RT dose for primary tumor	60 Gy (36–68 Gy)	62 Gy (36–70 Gy)	NS
RT dose for lymphatics	46 Gy (0–56 Gy)	50 Gy (36–54 Gy)	NS
Dose of parotid gland (right)	49.7 Gy	28.5 Gy	0.025
Dose of parotid gland (left)	48.3 Gy	28.3 Gy	0.023
Dose of submandibular gland (right)	59.1 Gy	52.3 Gy	NS
Dose of submandibular gland (left)	58.15 Gy	50.2 Gy	NS
Dose of pilocarpine (initial)	15 mg/day	10 mg/day	NS
Dose of pilocarpine dose (maintenance)	10 mg/day	10 mg/day	NS
Duration of pilocarpine medication	25 month (6–36 months)	12 month (3–16 months)	0.045

*3DCRT* 3D conformal radiotherapy, *IMRT* intensity-modulated radiotherapy

## Discussion

Radiotherapy-induced dry mouth is frequently observed as early and late side effects of radiotherapy. Advances in modern technology allow for the reduction of doses received by the parotid gland; however, submandibular and other minor glands are generally involved in the treatment target volumes [2, 7, 8, 10]. Amifostine is the only proven radioprotectant, and its higher cost and side effects have limited the widespread adoption [14–18]. Thus, non-technological solutions such as radioprotectants and symptomatic medications like pilocarpine may continue to be considered in the IMRT era. On the other hand, the medications have been particularly applied in follow-up period rather than concurrent usage to achieve symptomatic improvement [31]. However, concerns persist, particularly with pilocarpine, where frequent and severe side effects at recommended doses may necessitate patient discontinuation. Consequently, alternative practical forms such as spray, topical, or lozenge formulations have been developed [32–34].

In our study, as pilocarpine is only available in oral form in our country, we prospectively observed patients receiving systemic pilocarpine through self-reported symptom monitoring. Pilocarpine was prescribed in low doses (5 mg/day) and titrated based on patient necessity and toxicity. Despite experiencing dry mouth and sticky saliva in grade 2 or more as late toxicity, the advantage of a lower medication dose was evident, with only 16.6% of patients discontinuing due to toxicity-related discomfort. Thus, consideration of its use in patients who can tolerate lower doses may be practical.

Dosage has been discussed in various studies. In a phase III study, 15 mg/day was found tolerable [31]. Another study by Rieke et al. [35] demonstrated the safety of pilocarpine, even in doses up to 15 mg three times a day. Considering our previous clinical experience and the challenges faced by patients in adhering to high doses, we opted to initiate treatment with lower doses to balance patient needs and side effects. Notably, those discontinuing pilocarpine mostly used the drug in low doses.

Moreover, in patients undergoing IMRT, complete saliva protection may not be achievable due to submandibular gland doses. Thus, we recommend pilocarpine for patients where minor salivary glands are encompassed in high-dose volumes, advocating for medical symptomatic treatment despite the IMRT technique. Johnson et al. [21] reported the improvement in symptoms with post-RT 5 mg/day pilocarpine in their study that the patients were irradiated with 3DCRT technique. For IMRT patients, lower daily dose of pilocarpine may be considered if the high-risk doses will be ordered to nasopharynx, oral cavity, and oropharynx.

General quality of life questionnaires is not asked in depth about specific symptoms in the head and neck. For this reason, a disease-specific questionnaire specific to dry mouth is recommended as PROMs for evaluation of symptoms [36]. In this study, we prefer to use the questionnaire that we used before [27]. Meanwhile, in a systemic review that argues the PROMs quality, validity, and adequacy, none of the PROMs was found to adequately meet salivary gland dysfunction in terms of symptom severity or quality of life [37]. This is one of the weak points of our study.

It may be difficult to explore deeply into specific symptoms in the head and neck region with general quality of life questionnaires. Therefore, it is advisable to utilize a disease-specific questionnaire focused on dry mouth as Patient-Reported Outcome Measures (PROMs) for a thorough evaluation of symptoms [36]. In our study, we chose to employ the same questionnaire utilized in our previous research [27].

A systematic review assessing the quality, validity, and adequacy of various PROMs for salivary gland dysfunction found that none sufficiently addressed symptom severity or quality of life [37]. This represents a limitation in our study, as the selected questionnaire may not comprehensively capture the impact of dry mouth on patients. We acknowledge this as a weakness in our methodology and recommend that future research explores and develops more precise and comprehensive PROMs specifically tailored to assess salivary gland dysfunction.

While not statistically significant, the literature has reported taste improvement in patients following pilocarpine [38]. In our study, we observed improved taste quality during and after the medication in some of our patients. In addition, we noted enhanced sleep quality with patients reporting nighttime comfort without waking up for water or carrying a water bottle during the day, consistent with findings in the previous studies [39]. In addition, considering that head and neck cancer patients are generally older adults with prevalent smoking-related comorbidities, lower dose administration may aid in managing pilocarpine’s side effects.

## Conclusion

In systemic reviews and evidence-based guidelines, pilocarpine usage for radiotherapy-induced dry mouth in head and neck cancer patients has been identified. However, the sources contributing to these data exhibit a high risk of bias and limited quality. Consequently, the recommendation of pilocarpine remains controversial in terms of the benefit-to-side effect ratio for radiotherapy patients experiencing xerostomia.

Our observations suggest that pilocarpine could be employed for an extended duration at doses lower than those recommended in prospectuses for late-term dry mouth in these patients. This approach proves particularly beneficial in cases where submandibular glands receive high radiation doses. With modern radiotherapy techniques effectively preserving the parotid gland, short-term use may be recommended in these patients.

Future studies should aim to enhance the development of a more robust patient selection criteria model. This would contribute to refining the understanding of pilocarpine’s efficacy and tolerability in specific subsets of head and neck cancer patients undergoing radiotherapy.
